# Practices of French General Practitioners Regarding Vaccination of Boys Against Human Papillomavirus (HPV), One Year After the Application of Its Official Recommendation

**DOI:** 10.1007/s13187-024-02407-y

**Published:** 2024-02-14

**Authors:** Aline Hurtaud, Alexandre Abou Tara, Leïla Bouazzi, Yannick Pacquelet, Marie Boiteux-Chabrier, Bach-Nga Pham, Hélène Pierre Cavard, Coralie Barbe

**Affiliations:** 1 Département de Médecine Générale, Université de Reims Champagne-Ardenne, UFR Médecine, 51 rue Cognacq Jay, 51100 Reims, France; 2 Comité Universitaire de Ressources pour la Recherche en Santé, Université de Reims Champagne-Ardenne, UFR Médecine, 51 rue Cognacq Jay, 51100 Reims, France

**Keywords:** Primary care, Vaccine, Human papillomavirus, Boys, General practitioners

## Abstract

In 2019, French health authorities extended the recommendation for human papillomavirus (HPV) vaccination to include boys aged 11 to 19 years. We describe HPV vaccination practices among French general practitioners (GPs) since this recommendation wasapplied. We also identified factors associated with the propensity to propose HPV vaccination to boys. Cross-sectional study, between May and August 2022, among French GPs using a questionnaire asking about the GPs, their practices, and opinions regarding HPV vaccination, including whether they systematically proposed HPV vaccination to eligible boys or not. We investigated factors associated with systematic proposal of HPV vaccination, using univariate and multivariate logistic regression. In total, 360 GPs participated (76.6% females; mean age 34.7 ± 7.8 years; 22.9% had additional training in gynecology or pediatrics); 5.5% reported that they systematically offered HPV vaccination to boys prior to the recommendation, whereas 61.2% do so systematically since the recommendation. Factors associated with systematic proposal to boys (post recommendation) were female GP sex (78.6% versus 66.2%; OR = 2.0 [95% confidence interval (CI) 1.2–3.3]; *p* = 0.007) and systematic proposal prior to the recommendation (8.5% versus 0.7%; OR = 13.3 [1.7–101.7]; *p* = 0.01). Protection against HPV-induced cancer was cited as an argument to vaccinate girls (98.3% versus 89.2%; *p* < 0.0001); while reducing the risk of transmission was more commonly an argument to vaccinate boys (78.1% versus 51.8%; *p* < 0.0001). This study underlines the positive impact of the official recommendation for HPV vaccination of boys on the attitude of GPs, with an increase in the systematic proposal of HPV vaccination to boys.

## Introduction

Infections caused by the human papillomavirus (HPV) are the most common sexually transmitted diseases in the world [1, 2]. The majority of men and women will be exposed to HPV at some point during their lifetime, mostly before the age of 45 years [1, 3]. There are over 200 types of HPV, of which 12 are considered to be high-risk or potentially oncogenic, and may cause cancer of the cervix, anus, vulva, vagina, penis, or the ear/nose/throat (ENT) area, especially the oropharynx [1, 2].

The majority of HPV infections are asymptomatic and clear spontaneously [2]. Nevertheless, in 2018, an estimated 690,000 new cases of HPV-induced cancers were diagnosed worldwide, of which 69,400 occurred in men [1]. In France in 2015, there were an estimated 6333 new cases of HPV-induced cancer (all localizations) [4]. While women are more frequently affected by HPV-induced cancers, men may also be affected, especially localizations such as the anus, ENT area (oral cavity/oropharynx), and penis [4].

Since July 2007, HPV vaccination has been an integral part of the standard vaccine schedule for girls aged 11 to 14 years in France and is reimbursed by the national healthcare system for this purpose. Catch-up vaccination is also possible between 15 and 19 years of age, for girls who did not complete the two-dose schedule [5]. In 2020, the HPV vaccination uptake among girls aged 15 was reported to be 29.4% [6], which is far from the objective of 60% laid down by the national cancer plan for the 2014–2019 period [7]. At the European level, numerous countries recommend HPV vaccination for both boys and girls, as do the USA (since 2011) and Australia (since 2013) [8]. In this context, in December 2019, the French national health authority (Haute Autorité de Santé, HAS) issued a recommendation to extend HPV vaccination to boys, based on the assumption that with sufficient uptake, vaccination of boys would make it possible to mitigate the spread of HPV in the general population. This would yield improved protection for boys and men, regardless of their sexual orientation, but also for unvaccinated girls and women [8]. This recommendation became applicable in 1 January 2021 with HPV vaccination reimbursed under the national healthcare system for both boys and girls aged 11 to 14 years, with catch-up vaccination possible from 15 to 19 years [9].

General practitioners (GPs) are on the front line in promoting HPV vaccination and therefore need to adapt their practices to the new recommendations. Derhy et al. showed in a previous study that the primary source of information for parents about HPV vaccination is the GP, with 86% of parents citing the GP as their main source [10]. In 2019, a survey performed in a representative sample of 300 GPs by the National Cancer Institute (InCA), and HAS showed that 84% of respondents would recommend HPV vaccination to boys if it was integrated into the standard vaccine schedule [10].

The aim of this study was, therefore, to describe practices among GPs in France in terms of HPV vaccination after the application of the recommendation to vaccinate boys and to identify factors associated with the propensity to offer HPV vaccination to boys.

## Methods

### Study Design and Population

We performed an observational, cross-sectional study in France between 25 May and 11 August 2022, via a questionnaire disseminated widely via social networks and also through the Council of Physicians at the departmental level. The target population was GPs practicing in France, including those working as locums. In France, GPs’ locums are qualified GPs who have not yet set up a practice or non-qualified students with at least 18 months of medical experience. Due to the descriptive purpose of the study, no calculations were made regarding the sample size.

### Data Recorded

The questionnaire was drawn up in consultation with a team of GPs and methodologists and contained three parts. The first part recorded the GP’s socio-demographic characteristics, namely sex, age, form of practice, whether or not they had any additional training in gynecology and/or pediatrics, and whether or not they currently had any patients (men or women) who were being followed for HPV-induced complications or cancer. The second part investigated the GPs’ professional practices in terms of HPV vaccination, asking about whether or not they proposed HPV vaccination to boys and girls before the application of the new recommendation; whether or not they proposed HPV vaccination to boys and girls since the application of the new recommendation; on which occasion(s) they propose this vaccination; the arguments they use to encourage HPV vaccination in boys and girls; and acceptance of HPV vaccination among boys, girls, and the parents (of boys and/or girls). The third part of the questionnaire asked about the GPs’ own opinions about HPV vaccination, including their opinion regarding vaccination in general; their opinion about HPV vaccination for girls; their opinion about the expansion of the indication for HPV vaccination to include boys; their opinion about the 9-valent vaccine (Gardasil®9, Merck), the impact of the non-mandatory status of HPV vaccination, their opinion about the possibility of a vaccination campaign in schools, or of a dedicated consultation with a specific billing code. The time required to complete the questionnaire was around 5 min.

### Ethical Considerations

The LimeSurvey® platform was used to create and house a database containing the study responses at the University of Reims Champagne-Ardenne, France. All data were anonymous. Data were managed in accordance with the General Data Protection Regulations and French legislation concerning data privacy. As the study was based on an analysis of professional practices, based on anonymized data, and purely observational, it was exempt from Institutional Review Board approval according to the French Public Health Code (L1121-1, law 2012–300, March 5, 2012). The study was registered with the public database of healthcare projects Health Data Hub under the number F20230104100800.

### Statistical Analysis

All questionnaires were used in the statistical analysis, whether complete or incomplete. Considering the descriptive nature of the study’s main objective and a low number of missing data (no more than 5% of data), no data imputation was performed.

Quantitative variables are described as mean ± standard deviation, when normally distributed, or median and interquartiles if non-normally distributed. Categorical variables are described as number and percentage. GPs were classified into two groups, according to how often they propose HPV vaccination to boys since the application of the recommendation by the French Health Authorities, namely systematically vs often/sometimes/never. Continuous variables were compared between groups using the Student *t* or Mann–Whitney test as appropriate, and categorical variables using the chi-square or Fisher’s exact test as appropriate. Factors associated with vaccination with a *p*-value < 0.20 by univariate analysis were included in a multivariate logistic regression model, using backward selection, with an exit threshold set at 0.05. Results are expressed as odds ratios with 95% confidence intervals (CI). All analyses were performed using SAS version 9.4 (SAS Institute Inc., Cary, NC) and a *p*-value < 0.05 was considered statistically significant.

## Results

A total of 360 GPs participated in the study. The characteristics of the participants are detailed in Table 1. The majority were female (259/352, 76.6%) and mean age was 34.7 ± 7.8 years. More than half were locums (182/360, 50.6%). More than one-third worked in group practices or pluridisciplinary health centers (135/360, 37.5%). Fifty-nine GPs (16.4%) had additional training in gynecology and 25 (6.9%) in pediatrics. The majority had female patients who were being followed for HPV-related complications or cancers (279/354, 78.8%), while a minority (70/354 19.8%), had male patients being followed for the same pathologies.

**Table 1:** Description and opinions of participating French general practitioners regarding vaccination (*N* = 360)

	*N* = 360
Sex^a^	
- Female	259 (76.6)
- Male	93 (26.4)
Age, mean ± SD, years	34.7 ± 7.8
Type of practice	
- Works alone in own practice	28 (7.8)
- Works in a group practice or pluridisciplinary health center	135 (37.5)
- Locum	182 (50.6)
- Employee	20 (5.5)
Additional training	
- Gynecology	59 (16.4)
- Pediatrics	25 (6.9)
Follow female patients for HPV-related cancer or complications^a^	279 (78.8)
Follow male patients for HPV-related cancer or complications^a^	70 (19.8)
Opinion regarding vaccination (all vaccines, all patients)^a^	
- Strongly opposed	0
- Somewhat opposed	1 (0.3)
- Somewhat in favor	34 (9.8)
- Strongly in favor	311 (89.9)
- No opinion	0
Opinion regarding HPV vaccination for girls aged 11 to 19^a^	
- Strongly opposed	0
- Somewhat opposed	1 (0.3)
- Somewhat in favor	29 (8.4)
- Strongly in favor	314 (91.0)
- No opinion	1 (0.3)
Opinion regarding HPV vaccination for boys aged 11 to 19^a^	
- Strongly opposed	2 (0.5)
- Somewhat opposed	0
- Somewhat in favor	47 (13.6)
- Strongly in favor	293 (84.9)
- No opinion	3 (0.9)
Have doubts about the efficacy of Gardasil®9^a^	5 (1.4)
Have doubts about the safety of Gardasil®9 (possible side effects)^a^	4 (1.2)
In favor of a school-based HPV vaccination campaign^a^	271 (79.2)
In favor of a dedicated HPV vaccine consultation for adolescents^a^	227 (66.4)
Sources of information about the expansion of the recommendation for HPV vaccination to boys^a^	
- Public health authorities	103 (29.9)
- Specialized websites	87 (25.2)
- Professional network	281 (81.4)
- Personal network	105 (30.4)
- Programs for lay people (television, radio) and/or social networks	17 (4.9)
- Medical journals	9 (2.6)

*SD* standard deviation, *HPV* human papillomavirus

^a^Results were calculated on available data

The GPs’ opinions about vaccination are reported in Table 1. GP were generally strongly in favor of vaccination in general, and HPV vaccination in girls. The majority were also in favor of the expansion of vaccination to boys aged 11 to 19 years, with only 2/345 GPs (0.5%) reporting that they were opposed to the measure. Regarding the 9-valent vaccine, there were few doubts about its efficacy or safety, with only 1.4% of GPs expressing doubts about efficacy, and 1.2% expressing concerns about potential side effects.

More than three-quarters of GPs (271/342, 79.2%) were in favor of implementing vaccination campaigns in schools, and 66.4% (227/342) were in favor of a dedicated consultation about vaccination, with a specific billing code. The main source of information about the extension of the HPV vaccination to boys was the GPs’ professional network (281/345, 81.4%).

The respondents’ practices in terms of HPV vaccination are detailed in Table 2. Before the application of the expansion of the recommendation, HPV vaccination was systematically proposed to girls by 84.7% of respondents (293/346), whereas 5.5% (19/345) proposed it systematically to boys, and 53.6% (185/345) never propose it to boys at all. Since the application of the new recommendation, 61.2% (211/345) reported systematically proposing the HPV vaccination to boys, and 2.6% (9/345) never proposing it at all. The respondents indicated that they most often proposed HPV vaccination at the time of the scheduled immunization at 11 years of age (328/351, 93.4%), during routine consultations (295/351, 84.3%), during consultations to establish medical certificates for sports activities (269/351, 76.6%) or during consultations with the parents (93/351, 26.5%). The arguments reportedly used by the GPs to promote HPV vaccination were not the same for boys and for girls. Protection against HPV-induced cancers was more frequently used as an argument for vaccinating girls (345/351, 98.3% versus 313/351, 89.2%; *p* < 0.0001), whereas reducing the risk of transmission in the general population was more commonly used as an argument for vaccination of boys (274/351, 78.1% versus 182/351, 51.8%; *p* < 0.0001). Adherence to HPV vaccination was easily obtained for 96.1% of girls, 91.1% of girls’ parents, 76.8% of boys, and 69.7% of boys’ parents.

**Table 2:** Respondent GPs’ professional practices in terms of HPV vaccination

	*N* = 360
Proposed HPV vaccination to boys aged 11 to 19 before the expanded recommendation from the health authorities in 2021^a^	
- Systematically	19 (5.5)
- Often	43 (12.5)
- Sometimes	98 (28.4)
- Never	185 (53.6)
Propose HPV vaccination to boys aged 11 to 19 since the expanded recommendation from the health authorities in 2021^a^	
- Systematically	211 (61.2)
- Often	102 (29.6)
- Sometimes	23 (6.7)
- Never	9 (2.6)
Propose HPV vaccination to girls aged 11 to 19^a^	
- Systematically	293 (84.7)
- Often	45 (13.0)
- Sometimes	7 (2.0)
- Never	1 (0.3)
When do you propose HPV vaccination?^a^	
- During routine consultations	296 (84.3)
- At the time of scheduled immunization at 11 years of age	328 (93.4)
- When writing medical certificates for sports activities	269 (76.6)
- When the parents consult for other motives	93 (26.5)
- Don’t propose vaccination	3 (0.8)
Arguments used to encourage HPV vaccination in girls^a^	
- Protection against HPV-related cancers	345 (98.3)
- Protection against genital warts	199 (56.7)
- Reduce risk of transmission in the general population	182 (51.8)
- Cite example of positive results obtained in other countries	227 (64.7)
- Cite the health authority guidelines	102 (29.1)
- 65% of the cost of vaccine reimbursed by national health insurance	59 (16.8)
- Don’t propose vaccination	1 (0.3)
Arguments used to encourage HPV vaccination in boys^a^	
- Protection against HPV-related cancers	313 (89.2)
- Protection against genital warts	230 (65.5)
- Reduce risk of transmission in the general population	274 (78.1)
- Cite example of positive results obtained in other countries	211 (60.1)
- Cite the health authority guidelines	107 (30.5)
- 65% of the cost of vaccine reimbursed by national health insurance	71 (20.2)
- Do not propose vaccination	7 (2.0)

*HPV* human papillomavirus

^a^Results were calculated on available data

By multivariate analysis, the factors associated with systematic proposal of HPV vaccination to boys since the application of the new recommendation by the French health authorities were female sex of the GP (OR 2.0 (95%CI 1.2–3.3), *p* = 0.007), and having been in the habit of proposing HPV vaccination systematically prior to the new recommendation (OR 13.3, (95%CI 1.7–101.7), *p* = 0.01) (Table 3).

**Table 3:** Factors associated with systematic proposal of HPV vaccination to boys by GPs since the introduction of the recommendation in 2021

	Systematically offer HPV vaccine to boys since 2021		Univariate analysis	Multivariate analysis	
	Yes (*N* = 211)	No (*N* = 141)	*P*	OR (95% CI)	*P*
Female sex^a^	165 (78.6)	88 (66.2)	0.01	2.0 (1.2–3.3)	0.007
Age, mean ± SD, years	34.9 ± 7.3	34.3 ± 8.5	0.44	-	-
Work in pluridisciplinary health center^a^	91 (43.1)	42 (31.3)	0.03	-	-
Additional training in gynecology and/or pediatrics^a^	44 (20.8)	27 (20.1)	0.19	-	-
Follow male patients for HPV-related cancer or complications^a^	50 (23.7)	20 (14.9)	0.08	-	-
Follow female patients for HPV-related cancer or complications^a^	175 (82.9)	97 (72.4)	0.06	-	-
Systematically offered HPV vaccination to boys aged 11 to 19 prior to a new recommendation	18 (8.5)	1 (0.7)	0.002	13.3 (1.7–101.7)	0.01
Doubts about HPV vaccine efficacy^a^	1 (0.5)	4 (3.0)	0.06	-	-
Doubts about HPV vaccine safety (potential side effects)^a^	1 (0.5)	3 (2.3)	0.13	-	-

*SD* standard deviation, *OR* odds ratio, *CI* confidence interval

^a^Results were calculated on available data

## Discussion

More than 1 year after the application of the recommendation of the French health authorities extending the HPV vaccination to boys, our study describes the practices of French GPs in terms of HPV vaccination, particularly in boys. We report that 84.6% of GPs were in favor of vaccinating boys against HPV. The proportion of GPs that systematically offer HPV vaccination to boys has increased markedly rising from 5.5 to 61.2%. These findings are in line with the literature. Another French study, published in 2023, found that 70% of GPs were in favor of HPV vaccination in boys, and 63.2% reported that they systematically offered it to boys since the new recommendation was introduced [11]. In a qualitative study, Bouchez et al. showed that the existence of an official recommendation and professional consensus about the need to vaccinate boys against HPV were elements that prompted physicians to offer HPV vaccination to boys [12]. Elsewhere, in a study among 357 physicians in Malaysia, Wong et al. reported that a lack of guidelines from the health authorities regarding the recommendation of HPV vaccine was cited as one of the main reasons for non-recommendation [13].

In our study, the majority (84.7%) of GPs systematically recommended HPV vaccination to girls. The expansion of the indication to include boys had the corollary effect of increasing coverage among girls, which reached 43.6% in metropolitan France in 2021 [14]. In a survey performed in 2019, the French National Cancer Institute (InCA) and National Health Authority (HAS) found that 88% of physicians did not systematically recommend HPV vaccination to girls, declaring that they would be more inclined to do so if the recommendation also included boys [10]. More than two-thirds of GPs in that survey further declared that the main lever for improving uptake of HPV vaccination among girls would be to recommend the vaccine for boys also (68%) [10].

The majority of GPs in our study (79.2%) also declared that they were in favor of a vaccination campaign in schools. School-based vaccination is an effective means of reaching the target population, because the targets for vaccination can be easily identified, and are all situated together in the same location, with limited logistical constraints [15]. This approach is also particularly advantageous among adolescents, who may otherwise have less frequent contact with GPs than younger children [16]. A study from Australia reported a positive impact of school vaccination campaigns, making it possible to inform about the vaccine, and positively influence the parents’ decisions [16]. In this context, the French government has rolled out a HPV vaccination campaign for consenting adolescents in their second year of secondary school (i.e., age 12), for the 2023–2024 academic year [17]. In our study, the GPs also reported that they would like to see the creation of a dedicated consultation about vaccination, with a specific billing code to enable reimbursement. Since early 2022, a new billing code has been available in the French healthcare system covering “First consultation for contraception and prevention in sexual health” [18]. It can occur once per patient, men or women up to the age of 25, with no minimum age, and already encompasses explanations about the different types of contraception available, or how to prevent sexually transmitted infections. It is currently billed at 47.5 Euro (26.5 Euro for a standard consultation) and is fully reimbursed by the national healthcare system with a total advance of the expenses. Given that HPV vaccination can prevent the spread of HPV, known to be responsible for cervical cancer and genital warts, this billing code could be used to promote HPV vaccination in adolescents but seems less appropriate at the age of 11 to 14 years.

In our study, the propensity to systematically propose HPV vaccination to boys since the application of its recommendation was strongly linked to the GP’s prior habit of systematically proposing it before that. This shows that GPs who were already convinced of the utility of HPV vaccination for boys before the indication was extended to cover them have remained firm advocates since the recommendation was applicated. Habermacher et al. previously showed that physicians who were strongly in favor of extending the indication for HPV vaccination to boys proposed it significantly more frequently (adjusted odds ratio 12.5, 95%CI 8.3–20) [11]. Furthermore, in our study, only a very small proportion of respondents expressed doubts about the efficacy (1.4%) or safety (1.2%) of the HPV vaccine. These results should be put in perspective with an Italian study from 2021 reporting that GPs were more inclined to propose HPV vaccination to boys aged 11 to 12 when they believed the vaccine to be efficacious and safe for the prevention of HPV-induced complications in men [19]. In our study, female GPs were significantly more likely to systematically propose HPV vaccination to boys since the introduction of the new recommendation. This may be explained by a lack of knowledge among male GPs about HPV, compared to their female peers [20]. Indeed, in this regard, Allison et al. also found that male physicians were less likely to have HPV vaccine discussions (adjusted odds ratio for having such discussions “rarely or never” 1.8, 95%CI 1.1–3.1) [21].

We noted that the arguments put forth by GPs to encourage vaccination were not the same, depending on whether the potential vaccinee was a girl or a boy. The argument that HPV vaccination prevents HPV-induced cancers was more frequently used to inform and encourage girls, whereas the reduced risk of HPV transmission in the general population was more frequently used with boys. This finding is also congruent with the study of Habermacher et al., where the leading argument cited for encouraging boys to accept HPV vaccination was indirect protection of girls against cervical cancer by limiting sexual transmission (84.3%), followed by the argument of self-protection from HPV-induced oropharyngeal, anal, and penile cancers (74.5%) [11].

Finally, our study also shows that acceptance of HPV vaccination is higher among girls and their parents than among boys and their parents, according to the respondent GPs. In 2019, a study of HPV vaccine attitudes and decision-making in 1049 parents in England and Wales also found that parents of boys more frequently had information needs about HPV vaccine than parents of girls, and girls’ parents were more willing to vaccinate their child compared to parents of boys (adjusted odds ratio 1.80, 95%CI 1.32–2.45) [22].

The main limitation of our study is the mode of distribution of the questionnaire, which was mainly disseminated on social networks, thus leaving some margin for selection bias. Indeed, physicians who are more engaged and interested in vaccination may be more likely to participate. Our population of respondents is also younger than the average of GPs in France (34.7 ± 7.8 years old on average in our study, compared to a mean age of 50.3 years nationally) [23].

Many implications could be derived from these findings. First, as male GPs do not propose the HPV vaccination to boys as often as female GPs do, it could be useful to target medical courses for men GPs with specific messages able to encourage them to promote HPV vaccination for boys. This implies to understand beforehand what are the determinants of a lesser motivation in male GPs to propose the HPV vaccination to boys. It could also be interesting to study to what extent the medical argument in favor of the protection against HPV-induced cancers would be more effective to promote HPV vaccination in boys, instead of the argument in favor of reducing the transmission to others that is currently used with them. Secondly, even if consistent results of studies show that French GPs are largely in favor of the HPV vaccination for girls and boys, this positive opinion seems to be not enough to reach the objectives of vaccine coverage fixed by the French health authorities: 60% in 11–19-year-old adolescents in 2023 and 80% about 2030 [24]. Currently, the vaccine coverage for HPV in France is one of the weakest in the industrialized countries, even though coverage in 2022 or 2023 is not yet available: only 45.8% of 15-year-old girls and under 6% of boys had had one dose of HPV vaccine [25]. This insufficient coverage for HPV vaccination refers obviously to the strong global vaccine reluctance known among the French population [26]. The HPV vaccination was also the only non-obligatory vaccination in children proposed in the French vaccination schedule (until the recent recommendation in favor of vaccination against meningitis B in infants). Finally, the fact that the extension of HPV vaccination in boys applied from the beginning of 2021, at the same time than the first Covid-19 ARN-vaccines arrived and all the questions about their security occurred, may have led more parents to refuse or delay HPV vaccination for their child. In this complex context, further research will aim to assess the impact of these contextual elements and identify the levers for action in favor of HPV vaccination in both girls and boys.

## Conclusion

One year after the application of the recommendation for HPV vaccination in boys, our study shows that this guideline had a positive impact on the practices of French GPs, with an increase in the systematic proposal of HPV vaccination to boys compared to practices before the recommendation. Respondent GPs were largely in favor of school-based vaccination campaigns, which could be an attractive means of increasing HPV vaccine coverage in France. Specific training of male GPs and a better choice of the arguments put forward in favor of HPV vaccination in boys could be implemented.

## Data Availability

The datasets used and/or analysed during the current study are available from the corresponding author on reasonable request.
